# An Unobtrusive and Calibration-free Blood Pressure Estimation Method using Photoplethysmography and Biometrics

**DOI:** 10.1038/s41598-019-45175-2

**Published:** 2019-06-13

**Authors:** Xiaoman Xing, Zhimin Ma, Mingyou Zhang, Ying Zhou, Wenfei Dong, Mingxuan Song

**Affiliations:** 1 Suzhou Institute of Biomedical Engineering and Technology, Chinese Academy of Sciences, Suzhou, Jiangsu 215163 China; 2 The Affiliated Suzhou Hospital of Nanjing Medical University, Suzhou Science and Technology Town Hospital, Department of Endocrinology, Suzhou, Jiangsu 215153 China; 3 First Hospital of Jilin University, Cardiovascular Department, Changchun, Jilin 130021 China; 4 Suzhou GK Medical Co. Ltd, Suzhou, Jiangsu 215163 China

**Keywords:** Translational research, Hypertension

## Abstract

We introduce a novel paradigm to unobtrusively and optically measure blood pressure (BP) without calibration. The algorithm combines photoplethysmography (PPG) waveform analysis and biometrics to estimate BP, and was evaluated in subjects with various age, height, weight and BP levels (n = 1249). In the young population (<50 years old) with low, medium and high systolic blood pressures (SBP, <120 mmHg; 120–139 mmHg; ≥140 mmHg), the fitting errors are 6.3 ± 7.2, −3.9 ± 7.2 and −20.2 ± 14.2 mmHg for SBP respectively; In the older population (>50 years old) with the same categories, the fitting errors are 12.8 ± 9.0, 0.5 ± 8.2 and −14.6 ± 11.5 mmHg for SBP respectively. A simple personalized calibration reduces fitting errors significantly (n = 147), and good peripheral perfusion helps to improve the fitting accuracy. In conclusion, PPG may be used to calculate BP without calibration in certain populations. When calibrated, it shows great potential to serially monitor BP fluctuation, which can bring tremendous economic and health benefits.

## Introduction

Hypertension is one of the most important risk factors for cardiovascular diseases. It has now become clear that daily BP readings have greater predictive power for cardiovascular events than isolated in-clinic measurements, because the later practice may introduce white-coat hypertension, masked hypertension, and ignores the BP variability^1^. Furthermore, although the accuracy of the commercial oscillometric devices is close to auscultation, they are bulky, slow and uncomfortable to use. Ambulatory BP assessment with cuffs is also intermittent and disturbs daily life.

Many technical innovations have been developed to monitor BP continuously and unobtrusively^2,3^, and the most promising one is PPG. PPG is an optical signal related to peripheral blood volume pulsations and its waveform has been proven to have a good correlation with BP waveform^4–6^.

Recent publications showed that PPG alone may be used to monitor BP continuously. In our previous work, we found that in continuous monitoring, PPG-derived BP achieved an accuracy of 0.06 ± 7.08 mmHg for systolic blood pressure (SBP), and 0.01 ± 4.66 mmHg for diastolic blood pressure (DBP) with initial calibration^7^. Watanabe *et al*. showed that calibration could be effective for much longer^8^. After one month, the intraclass correlation coefficient (ICC) between test SBP and reference SBP was 0.84, and ICC between test DBP and reference DBP was 0.75. However, there hasn’t been enough evidence to support a calibration-free BP estimation with PPG signals only. Shin *et al*. tested 25 normotensive subjects and obtained R = 0.818 for SBP^9^. Ruiz-Rodriguez *et al*. compared PPG-derived BP with invasive BP measurement in critically ill patients, and obtained an accuracy of −2.98 ± 19.35 mmHg for SBP, and −3.65 ± 8.69 mmHg for DBP, which limits its use in ICU, and could not replace traditional sphygmomanometers in the clinic^10^. Raichle *et al*. evaluated the performance of a BP smartphone app and found that it failed the accuracy test on 32 pregnant women^11^, which discredited the use of natural light to measure BP optically and questioned its validity in pregnant women.

It’s understandable. PPG-based techniques don’t really “measure” the pressure. Instead, they use the waveform feature analysis and theoretical models to predict the hemodynamics and link them to BP. However, the waveform may be easily influenced, and the correlation between peripheral pulsation and BP may not be optimal^12^. For example, Hashimoto *et al*. found that B:A ratio and aging index (AGI) derived from the second derivative of the finger photoplethysmogram (SDPTG) waveform is significantly correlated with SBP^13^. But finger sizes have a wide distribution and the pressure applied to the fingers are hard to control, which greatly affects the PPG waveform and reduces the BP estimation accuracy^14^. Cold temperature or poor circulation caused by diseases can also reduce the correlation between peripheral pulsation and blood pressure^15,16^. High blood viscosity slows down blood flow and considerablely influences the PPG waveform^17^. Hypertension may also be accompanied by diabetes, arrhythmia or pregnancy^18–22^, which may introduce unknown parameters to the model and deteriorate the fitting accuracy. Most importantly, the “volume” measured by PPG is actually the total amount of hemoglobin, which was assumed to be proportional to blood volume^23^. This assumption may fail in patients with anemia or edema. Therefore, careful consideration is warranted when selecting suitable patient groups to apply the new technology, so that optimal accuracy and reliability of readings can be ensured.

We hypothesize that biometric information could help reduce the uncertainty of PPG-based BP estimation. For example, body mass index (BMI) may be used to estimate contact pressures that applied to the measurement sites. Height combined with PPG waveform may be used to estimate blood flow velocity and blood vessel stiffness^24^. Age may be used to estimate pulse pressure (PP) range. Many previous publications either studied a few young and healthy subjects^9^ or used Multiparameter Intelligent Monitoring in Intensive Care (MIMIC) database^25–27^, which lacks demographic information. Thus, the goal of the present study is to cover subjects with diverse biometrics and develop a usable calibration-free algorithm. By implementing the most recent estimation scheme and pre-grouping the subjects, several measurements induced biases could be subtracted and fitting errors could be significantly reduced, which can be further improved by a larger training database and a personal calibration.

To address the agreement of PPG-derived BP with the reference BP, subanalyses were performed for

1. The young (≤50 years old) and the older (>50 years old) populations.
2. Normal (90 mmHg < SBP < 120 mmHg, group I), pre-hypertension and stage I hypertension (120 mmHg ≤ SBP < 140 mmHg, group II), stage II and stage III hypertension (SBP ≥ 140 mmHg, group III)^28^.
3. Low peripheral perfusion index (PI, <0.01) and high peripheral perfusion index (PI ≥ 0.01).
4. Comparison of calibrated and calibration-free BP estimation accuracy.

## Results

### BP estimation accuracy in the young and the older populations

In this study, the young population and the older population were considered separately, as due to considerable stiffness and hemodynamic characteristic differences between these two age groups. For example, the number of isolated hypertension cases increase with age, as shown in Supplementary Fig. 1.

**Figure 1:**
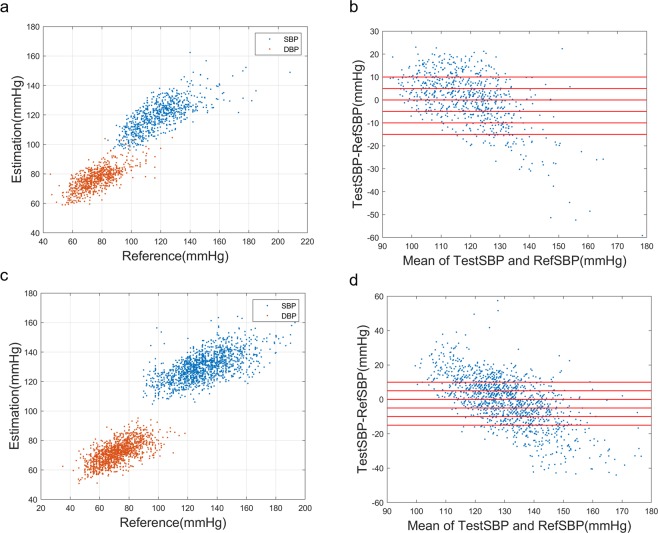
Scatter plot of estimated BP vs. reference BP for (**a**) the young group (**c**) the older group; Bland–Altman plot of testSBP vs. refSBP for (**b**) the young group (**d**) the older group.

A clear correlation was found between PPG-derived BP (testBP) and the reference BP (refBP), as shown in Fig. 1 and Table 1. However, the slope of fitting deviates from 1, which means low blood pressures are overestimated, and high blood pressures are underestimated. This problem is more serious in the older group, since some key features such as dicrotic notch may be gradually damped in aging and rigid blood vessels^29^, and more subjects have complications such as diabetes and hyperlipidemia. However, the fitting errors are mostly biases and can be significantly reduced with personal calibration or a prior knowledge of the SBP range.

**Table 1:** Calibration-free BP estimation: correlation with reference BP and the fitting errors in different age groups.

	Young Group (n = 739)	Older Group (n = 1340)
**Pearson Correlation Coefficient**		
R (SBP)	0.86	0.79
R (DBP)	0.83	0.81
**Fitting errors (mmHg)**		
SBP Error	0.45 ± 11.3	−0.68 ± 14.1
DBP Error	0.31 ± 8.55	−0.20 ± 9.0

n: measurement number.

We found that the calibration-free algorithm works best in the young groups with SBP lower than 140 mmHg, and the older group with SBP between 120 and 140 mmHg, as shown in Table 2. For older people, DBP fitting is more reliable than SBP. One interesting observation is that BP estimation accuracy is worse in the young hypertensive group than the older hypertensive group. This may be caused by unbalanced sample distribution. Since fewer young people have uncontrolled stage II and stage III hypertension, our model-based algorithm is less trained to distinguish hypertensive waveform in the young group.

**Table 2:** Calibration-free BP estimation: Fitting errors (mmHg) at different SBP levels.

	Group I	Group II	Group III
**Young**			
Measurements (n)	418	257	64
SBP Error	6.3 ± 7.2**	−3.9 ± 7.2	−20.2 ± 14.2**
DBP Error	3.6 ± 6.7**	−2.4 ± 7.3	−10 ± 11.7**
**Older**			
Measurements (n)	364	574	402
SBP Error	12.8 ± 9.0**	0.5 ± 8.2	−14.6 ± 11.5**
DBP Error	4.2 ± 7.0**	0.5 ± 7.8	−2.9 ± 8.9**

Group I: SBP < 120 mmHg; Group II: 120 mmHg ≤ SBP < 140 mmHg; Group III: SBP ≥ 140 mmHg.

**P < 0.001 versus group II.

### BP estimation accuracy in low PI and high PI groups

**Figure 2:**
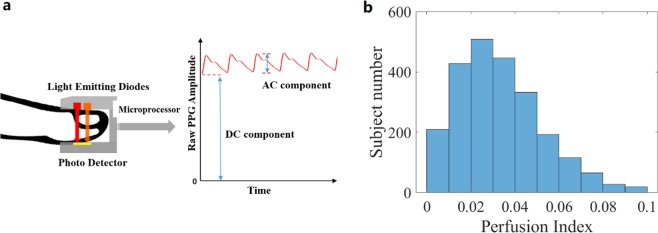
(**a**) Definition of perfusion index (**b**) PI distribution in test subjects.

The influence of peripheral perfusion on PPG-derived BP accuracy was also investigated. Peripheral PI was defined as the pulsating volume (AC) divided by the stationary volume (DC), as shown in Fig. 2. Intuitively, a better perfusion means a better correlation between branchial blood pressure and digital PPG. Our result confirmed the hypothesis, as shown in Table 3. One interesting observation is that for poorly perfused population, BP was underestimated. Poor PI means weaker pulses, and the external pressure has a larger influence on the microvascular blood vessels. As Grabovskis *et al*. pointed out, greater pressure leads to falsely high B:A ratio in SDPTG waveform, causing BP estimation to be lower^13,14^. This may be improved by warming up, pressure monitoring and initial calibration.

**Table 3:** Calibration-free BP estimation: Fitting errors (mmHg) at different PI levels

	PI < 0.01	PI ≥ 0.01
Young		
Measurement (n)	114	625
SBP Error	−2.8 ± 12.9*	1.1 ± 10.9
DBP Error	−1.6 ± 8.6*	0.7 ± 8.5
**Older**		
Measurement (n)	99	1241
SBP Error	−1.6 ± 15.5*	−0.6 ± 14.0
DBP Error	−2.7 ± 9.8*	0.0 ± 8.9

*P < 0.05 versus fitting errors when PI ≥ 0.01.

### Comparison of calibration-free and calibrated BP estimation accuracy

Although calibration-free BP estimation with PPG can’t replace the traditional sphygmomanometers, once calibrated, it could be useful in personal monitoring devices. To confirm findings by Watanabe *et al*.^8^, we followed 147 subjects for a month with one measurement per week. The biometric distribution of those subjects is summarized in Supplementary Table 1. A total of 505 usable measurements were collected, based on criteria described in the method section. The calibrated results are summarized in Table 4 and Supplementary Fig. 3. The calibration process was described in the method section and was supposed to reduce biometric related errors. We can see that the calibration process reduced biases, and significantly improved SBP fitting accuracy. DBP accuracy was significantly improved in the older group, and marginally for the young group (p = 0.09). We speculate that the majority of the DBP fitting errors for the young group may come from the PPG measurement, instead of the biometric estimation. The other reason may be the sample imbalance in our training set. In the young group, 85% of the pulse pressures (PP) are contained in 21.7–69.7 mmHg, while in the older group, 85% of the PPs are contained in 24.5–92.9 mmHg. The calibrated DBP accuracy in the young group may improve if a larger and more balanced database were used.

**Table 4:** Comparison of calibration-free and calibrated fitting errors (mmHg) for different age groups.

	Calibration-free	Calibrated
**Young**		
SBP Error	2.1 ± 13.6**	−0.1 ± 9.5
DBP Error	2.3 ± 9.5	−0.1 ± 9.0
**Older**		
SBP Error	5.5 ± 15.5**	0.0 ± 11.2
DBP Error	2.6 ± 9.3**	−0.2 ± 7.2

*P < 0.05 **P < 0.001 versus calibrated results.

## Discussion

Accumulated evidence has shown that PPG-based BP estimation may be used to monitor BP. This technological breakthrough helps us make extremely small, comfortable, noninvasive and inexpensive devices, which will facilitate its widespread application in continuous BP monitoring and cardiovascular health management. Although a completely calibration-free BP estimation with PPG may not be realistic for everyone, for a certain population, this technique may be used as a fast and easy screening tool for hypertension. With a simple personalized calibration, PPG-based BP estimation accuracy is significantly improved, which helps to make useful self-monitoring tools.

Despite its advantages, this technique should be used with caution. Special groups, such as children, pregnant women were not well-studied and were not included in this study. Since PPG signal is easily corrupted, BP during or immediately after exercise wasn’t evaluated. It’s hard to recruit young people with uncontrolled high BP, so our model may be substantially influenced by the data imbalance. For people with stage III hypertension, or arterial stiffness as may occur in the elderly, PPG waveform may lose some important features such as their dicrotic notches, thus the estimation may be less precise. When applying the technique, operators should know the limiting factors, and interpret the results correctly. In the future, more data should be collected to balance and improve the model. It’s also advisable to track subjects for several months to see what can influence the calibration factor, and how long the calibration factor can stay effective. This knowledge will help design more trustworthy personal health management products.

### Perspective

PPG is measured millions of times by pulse oximeters each day worldwide, however, only the signal amplitude is clinically used to extract blood oxygenation, which is a huge waste of its rich waveform information. Technically, if more clinicians are willing to record and share PPG data, as well as blood pressure information^30^, the size of this scientific database can expand rapidly. In this study, we found the main limiting factor for the model is the data size. This technology can only be truly ready when millions of data from various populations are taken, and a consensus is reached.

## Conclusion

We have developed a novel calibration-free BP estimation algorithm combining PPG waveform analysis and biometrics. Although this algorithm hasn’t met the requirement for medical use yet, our study shows a decent accuracy of PPG-derived BP for well-perfused, normal to the pre-hypertensive population. With initial calibration, BP estimation with PPG achieved better precision and shows potential as a personal BP monitoring technique.

## Methods

### Selection and description of participants

Both normotensive and hypertensive subjects were recruited from the local community (n = 661). If the same subject was measured on different days, it’s considered two measurements. To compare calibration-free and calibrated BP estimation accuracy, we invited a subgroup of the subjects (n = 147, from the local community) to complete a month-long study. Each subject was asked to come back one week later for another measurement. The demographic distribution of this subgroup is shown in Supplementary Table 1.

We also included data from 588 in-hospital subjects from Suzhou Science and Technology Town Hospital. Since PPG and BP data were taken as part of a normal medical procedure, we asked the permission of the patient to use the data anonymously. If the patient had more than one recording, we only took those on different days.

A total of 2358 measurements were recorded, and 279 measurements were excluded due to unstable hemodynamics or poor signal to noise ratio. A summary of the participant’s characteristics is shown in Table 5, and their detailed biometric distribution is shown in Supplementary Fig. 2.

**Table 5:** Subject characteristics.

	Community	In-hospital	Young Group	Older Group
Subject number	661	588	478	754
Measurement (n)	1140	1218	826	1532
Age (years)	59.1 ± 21.4	56.2 ± 16.4	36.0 ± 8.4	69.3 ± 11.6
BMI (kg/m^2^)	23.8 ± 3.4	24.8 ± 3.9	24.7 ± 4.2	24.1 ± 3.4
Height (cm)	162.8 ± 8.2	163.2 ± 8.6	166.8 ± 8.2	161.0 ± 7.8
SBP (mmHg)	125.5 ± 18.5	127.2 ± 19.3	118.7 ± 17.0	130.5 ± 18.6
DBP (mmHg)	71.9 ± 12.9	74.4 ± 11.6	76.2 ± 12.0	71.6 ± 12.2

### PPG and BP measurement procedures

In this study, the PPG waveform was measured by a validated medical pulse oximeter (BM2000A from Shanghai Berry Electronic Technology Co., Ltd), with a modified receiving protocol to record raw data. The examination site was on the index finger.

Subjects from the local community sat in an upright position with legs uncrossed and back supported. Their arms were supported on a flat table with the upper arm at heart level. Subjects were told to remain calm and motionless, and each subject was measured for at least 60 seconds. PPG was sampled at 50 Hz, and the raw data was transmitted to a cell phone and recorded in text files, which was later downloaded and analyzed. We inspected the 60 s data carefully to make sure that no drift or huge hemodynamic fluctuations occurred, as described in the method section. As a reference, BP was measured 2 minutes later by an electronic sphygmomanometer (MC6700 from Mindray) on the upper arm and 7 minutes later by an auscultatory sphygmomanometer (from Yuwell). The first cuff BP was designed to be measured shortly after PPG measurement to minimize the BP fluctuation. Since PPG measurement doesn’t interrupt the blood flow, it needs no time to recover. If the two reference values differ by more than 5 mmHg, the measurement was disqualified and not used in the following analysis. If not, we use the first BP value measured with the cuff as the reference. For in-hospital subjects, since their data were taken as part of a normal medical procedure, their reference BP was taken only once, within 5 minutes after their PPG measurement.

The purpose of this study is to collect as many data as possible to build a best-performing model, and evaluate its applicability. It’s not meant to validate a specific BP measuring device or algorithm, so we did not follow the standard validation procedure for BP measuring devices^31^.

### Data analysis

We propose a novel calibration-free BP estimation algorithm combining PPG and biometrics. An index of large artery stiffness^24^ was used to estimate pulse transition time, which requires the use of height information and PPG waveform analysis instead of the electrocardiogram. We also used BMI to adjust the algorithm for different contact pressures. Combined with an improved whole based PPG feature extraction^25^ and SDPTG feature extraction^8,27^, a random forest algorithm was developed to estimate BP. This combination is more stable and less prone to overfitting.

The detailed procedure was as follows:

#### Data selection

**Figure 3:**
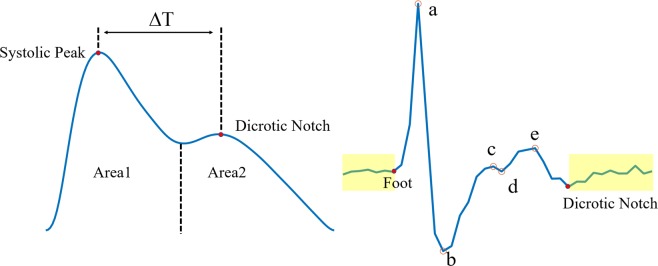
Definition of PPG waveform features (left) and definition of SDPTG features(right). Yellow boxes correspond to segments used to evaluate the noise level.

Since we only used one cuff-based BP reference per measurement, we’d like to keep the PPG measurement as stable as possible. An array of hemodynamic features was calculated on a beat to beat basis for each measurement, such as perfusion index, stiff index, heart rate, area 1 and 2 under the PPG waveform (as shown in Fig. 3). The stiff index was defined as *h*/$\triangle {\rm{T}}$, where *h* was the height of the subject, and $\triangle {\rm{T}}$ was the time lapse between systolic peak and dicrotic notch. If one feature from a particular beat was considered an outlier, this particular beat was labeled abnormal and discarded. If the percentage of abnormal beats for a measurement exceeded 20%, the entire measurement was excluded. The quality of SDPTG waveform was also very important, since we had to extract features correctly to train the model. The segment from a dicrotic notch to the next foot was used to evaluate the noise level, as shown in Fig. 3. If the standard deviation of this segment exceeded 15% of *b*, according to the definition of Takazawa *et al*.^32^, data from this particular beat was excluded.

#### Data preprocessing

BP was estimated on a beat-to-beat basis. One beat was defined to contain a complete cardiac cycle, and resampled to a 32-point array. A template was calculated for each measurement as the median of the PPG waveforms, and 30 cardiac cycles were selected from each measurement, based on the similarity to the template. If less than 30 good-quality cardiac cycles were available, the entire measurement was excluded.

#### Whole based feature extraction

A singular value decomposition (SVD) was performed to yield a matrix with a smaller dimension. For each PPG waveform, we chose 4 principal components that explained 95% of the variance, labeled as P_1–4_. We did the same decomposition to each SDPTG waveform, and used 8 components that explained 90% of the variance, labeled as S_1–8_. This method is more stable than the traditional feature extraction and is more easily implemented.

#### SDPTG-based feature extraction

Values of b/a, c/a, d/a and e/a values were also extracted as features according to the definition of Takazawa *et al*.^32^, as shown in Fig. 3.

#### The random forest algorithm

Each input array was composed of 19 features, including P_1–4_, S_1–8_, heart rate, b/a, c/a, d/a, e/a, SI and BMI. The training targets were set as SBP and DBP separately. Since only one set of SBP and DBP were available for each measurement, which included 30 sets of features, we added small perturbations to SBP and DBP to match the input numbers. A random number between −0.5 mmHg and 0.5 mmHg was chosen and added to each target value. For example, one measurement had 30 valid cardiac cycles, and the target SBP was 130 mmHg. For the training purpose, we generated 30 random numbers from 129.5 mmHg to 130.5 mmHg as the targets. A bagged regression tree algorithm was then used to generate a model, with the min leaf size set to 30.

#### Validation

The code was written with Matlab (Mathworks, Natick, MA). The validation was done by a leave-one-out procedure. Data from one subject was completely drawn out from the dataset as the test set. If the subject had multiple measurements, all the measurement data from this particular subject were excluded from the training set to avoid contamination. The rest of the data was trained to yield a model. Then the test set was evaluated by the trained model. To complete a leave-one-out validation for N subjects, this procedure has to run N times. This method mimics real calibration-free measurement and maximizes data usage.

### Personal calibration procedure

We hypothesized that the errors of PPG-derived BP were composed of two parts—one from the biometric adjustment and the other from the PPG signal. The first can be minimized by personal calibrations. The personal calibration factor was defined as the median of all his or her previous fitting errors using the calibration-free algorithm. Calibration of a new measurement was done by subtraction of the personal calibration factor.

### Human subjects research statement

This study conforms to the principles outlined in the Declaration of Helsinki and was approved by the ethics committee of Suzhou Science and Technology Town Hospital (algorithm development—approval number IRB2018043). All examinees provided informed consent before the measurements were conducted, in accordance with HIPAA regulations.

### Data Statistical Analysis

All statistical analyses were performed by using Matlab R2018a. To address the agreement of PPG-derived BP with the reference BP, scatter plots and Bland–Altman plots were created. Pearson correlation coefficient (R) between PPG-derived BP and the reference BP was also calculated. The fitting errors were assumed to be normally distributed and independent of each other. We performed a two-sample t-test on fitting error comparison of low PI and high PI, and calculated mean errors, the standard deviation (±SD) of errors, for different age groups separately. For comparison of calibrated and calibration-free fitting errors, we used a one-sided paired t-test to calculate the significance level. A value of P < 0.05 was taken as significant, and a value of P < 0.001 was taken as very significant.

## Supplementary information

Supplementary Information

## Data Availability

All data analyzed during the current study are available from the corresponding author on reasonable request.
