# Supplementary material for: An Unobtrusive and Calibration-free Blood Pressure Estimation Method using Photoplethysmography and Biometrics
Source: Sci Rep. 2019 Jun 13;9:8611. doi: 10.1038/s41598-019-45175-2 (PMC6565722; doi:10.1038/s41598-019-45175-2)
Supplement: Supplementary file 1 — Supplementary Information [file 41598_2019_45175_MOESM1_ESM.pdf]

An Unobtrusive and Calibration-free Blood Pressure Estimation Method using Photoplethysmography and Biometrics

Xiaoman Xing[1*+], Zhimin Ma[2+], Mingyou Zhang[3], Ying Zhou[2], Wenfei Dong[1]& Mingxuan Song[4]

1. Suzhou Institute of Biomedical Engineering and Technology, Chinese Academy of Sciences, Suzhou, Jiangsu, 215163, China
2. The Affiliated Suzhou Hospital of Nanjing Medical University, Suzhou Science and Technology Town Hospital, Department of Endocrinology, Suzhou, Jiangsu, 215153, China
3. First Hospital of Jilin University, Cardiovascular Department, Changchun, Jilin, 130021, China.
4. Suzhou GK Medical Co. Ltd., Suzhou, Jiangsu, 215163, China

* xingxm@sibet.ac.cn

[+] These authors contributed equally to this work.

These figures are intended for publication as an online data supplement.

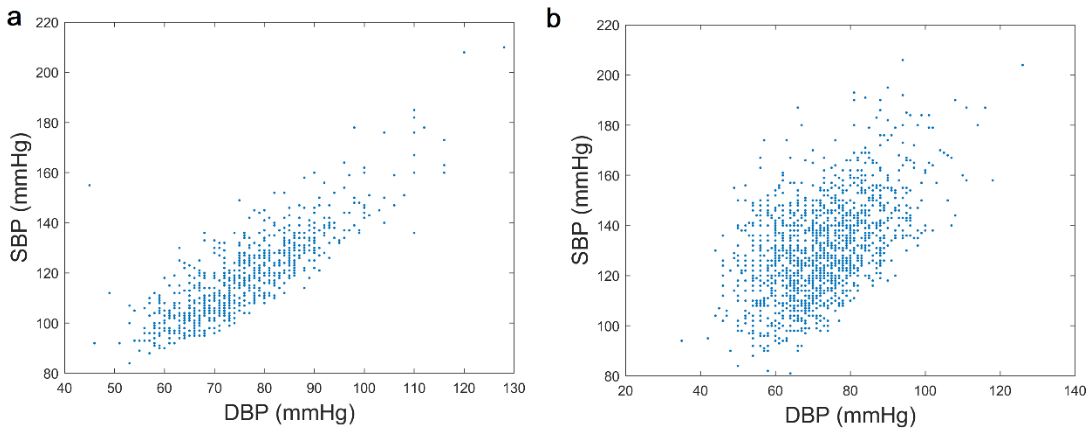

Figure 1. (a) SBP is significantly correlated with DBP in the young population (b) More isolated hypertension cases happen in the older population. SBP is less correlated with DBP.

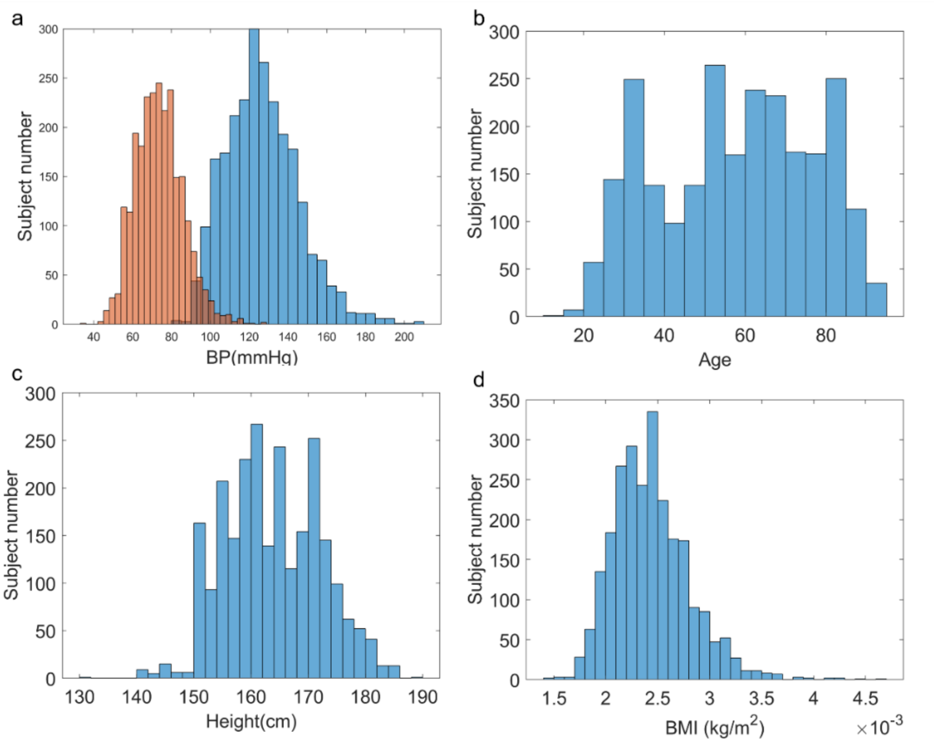

Figure 2. Distribution of participants based on (a) BP (b) age (c)Height (d)BMI.

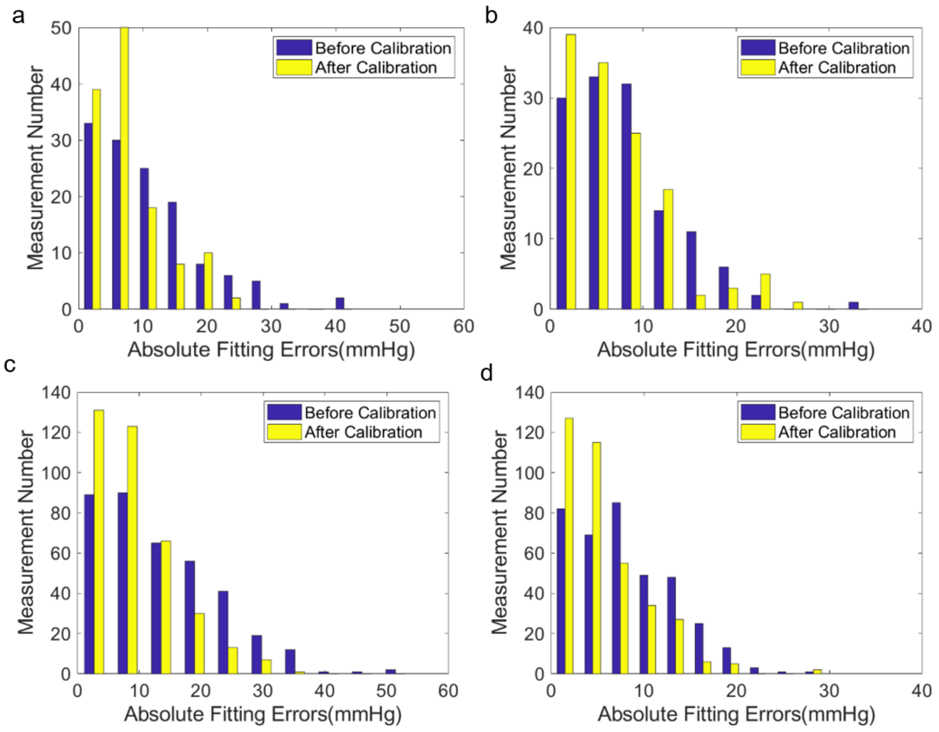

Figure 3. Fitting error comparison of calibration-free and calibrated algorithm for (a) SBP, the young group (b) DBP, the young group (c) SBP, the older group (d) DBP, the older group.

Table 1. Biometric distribution of follow-up subjects

	**Young Group**	**Older Group**
Subject number	40	107
Measurement number	129	376
Age	31.7±6.2	73.4±12.1
Height (cm)	168.8±7.8	159.6±6.8
BMI (kg/m^2)	23.1±3.7	24.2±3.3
SBP (mmHg)	114.6±16.2	121.3±17.3
DBP (mmHg)	72.3±10.8	73.1±10.3
